# Genetic resilience in chickens against bacterial, viral and protozoal pathogens

**DOI:** 10.3389/fvets.2022.1032983

**Published:** 2022-11-10

**Authors:** Haji Gul, Gul Habib, Ibrar Muhammad Khan, Sajid Ur Rahman, Nazir Muhammad Khan, Hongcheng Wang, Najeeb Ullah Khan, Yong Liu

**Affiliations:** ^1^Anhui Province Key Laboratory of Embryo Development and Reproduction Regulation, Anhui Province Key Laboratory of Environmental Hormone and Reproduction, School of Biological and Food Engineering, Fuyang Normal University, Fuyang, China; ^2^College of Animal Science and Technology, Anhui Agricultural University, Hefei, China; ^3^Department of Microbiology, Abbottabad University of Science and Technology, Abbottabad, Pakistan; ^4^Department of Food Science and Engineering, School of Agriculture and Biology, Shanghai Jiao Tong University, Shanghai, China; ^5^Key Laboratory of Animal Parasitology of Ministry of Agriculture, Laboratory of Quality and Safety Risk Assessment for Animal Products on Biohazards (Shanghai) of Ministry of Agriculture, Shanghai Veterinary Research Institute, Chinese Academy of Agricultural Sciences, Shanghai, China; ^6^Department of Zoology, University of Science and Technology, Bannu, Pakistan; ^7^Institute of Biotechnology and Genetic Engineering, The University of Agriculture, Peshawar, Pakistan

**Keywords:** chicken MHC, genetics, SNPs, non-coding RNAs, pathogens, infectious diseases, novel technology

## Abstract

The genome contributes to the uniqueness of an individual breed, and enables distinctive characteristics to be passed from one generation to the next. The allelic heterogeneity of a certain breed results in a different response to a pathogen with different genomic expression. Disease resistance in chicken is a polygenic trait that involves different genes that confer resistance against pathogens. Such resistance also involves major histocompatibility (MHC) molecules, immunoglobulins, cytokines, interleukins, T and B cells, and CD4+ and CD8+ T lymphocytes, which are involved in host protection. The MHC is associated with antigen presentation, antibody production, and cytokine stimulation, which highlight its role in disease resistance. The natural resistance-associated macrophage protein 1 (Nramp-1), interferon (IFN), myxovirus-resistance gene, myeloid differentiation primary response 88 (MyD88), receptor-interacting serine/threonine kinase 2 (RIP2), and heterophile cells are involved in disease resistance and susceptibility of chicken. Studies related to disease resistance genetics, epigenetics, and quantitative trait loci would enable the identification of resistance markers and the development of disease resistance breeds. Microbial infections are responsible for significant outbreaks and have blighted the poultry industry. Breeding disease-resistant chicken strains may be helpful in tackling pathogens and increasing the current understanding on host genetics in the fight against communicable diseases. Advanced technologies, such as the CRISPR/Cas9 system, whole genome sequencing, RNA sequencing, and high-density single nucleotide polymorphism (SNP) genotyping, aid the development of resistant breeds, which would significantly decrease the use of antibiotics and vaccination in poultry. In this review, we aimed to reveal the recent genetic basis of infection and genomic modification that increase resistance against different pathogens in chickens.

## Introduction

The breeding of chicks with polygenic resistance is the top priority of poultry farmers as these chickens may tolerate challenging environments without losing their productivity. The poultry industry is susceptible to bacterial, viral, and protozoal pathogens that cause several infectious diseases and reduce growth yield, productivity, and profit. Prophylactic measures, such as vaccination, antibiotics, disinfectants, and culling, are used to control infections in poultry (1). However, current vaccines lack cross-protection against multiple strains of each virus. Furthermore, the mutagenicity of viruses has led to the emergence of highly virulent strains (2). To counter emerging pathogens, a genetically resistant breed should be developed to prevent outbreaks, enable sustained economic viability, and retain consumer confidence in poultry products. By rearing genetically disease-resistant flocks, a breed that can withstand infectious diseases and pathogens owing to its unique genetic modifications, can be obtained (1, 3, 4).

Many disease-resistant genes, including MHC, chicken interleukin 1beta converting enzyme 1 (Caspase1), inducible nitric oxide synthase, IFN, Nramp-1, myxovirus-resistance gene, and toll-like receptor (TLR) genes, play a role in the active immune system of chickens (4, 5). The immune system varies among different hosts, which exhibit different responses to immune cells, such as T and B cells, antibody production, phagocytosis, and lymphocyte proliferation that protect the host from pathogen damage (3). The communication network of immune cells consists of T and B cell receptors, MHC, antibodies, and cytokines that are involved in antigen processing of the effector cells, and play a pivotal role in resistance and susceptibility against bacterial, viral, and parasitic diseases (3, 5). For instance, the Athens Canadian Random Bred strain, which is the oldest pedigreed meat-type chicken existing since the 1950's, has a more stable immune response and disease-resistant phenotype than modern-day broilers (6).

Based on genomic analysis, phosphoinositide-3-kinase–protein kinase B, Janus kinase/signal transducers and activators of transcription (JAK/STAT), nuclear factor kappa B (NF-κB), IL-1β, and IL-6 mRNA are highly expressed in Athens Canadian Random Bred compared to modern broiler (6). In our previous work, immunoglobulin lambda light chain precursor, Ig-gamma (clone-36 chicken), *P01875*, and *PIT-54* genes were identified to be involved in immune response during embryogenesis (7). In a subsequent study, dietary ellagic acid was found to significantly increase antioxidant and antibacterial activities in layers and improve bird health status (8). Importantly, breeding with new technologies improves poultry productivity and enhances disease resistance traits. For example, the livestock-breeding program produced nematode-resistant sheep (9). Similarly, birds resistant to lymphoid leucosis and Marek's disease (10), mastitis-resistant cattle (11), immunocompetent pigs (12), bird flu-resistant chickens (13), Trypanosoma resistant cows (14), porcine reproductive and respiratory syndrome virus-resistant pigs (15), and prion protein-resistant sheep and goat (16, 17) have been developed.

As poultry products are globally consumed on a large scale, there has been substantial interest in generating disease-resistant chicken. Here, we aimed to discuss the genetic responses of chickens to bacterial, viral, and protozoal pathogens, and summarize recent advancements in the generation of pathogen-resistant chickens *via* gene expression modulation using the CRISPR/Cas system (clustered regularly interspaced short palindromic repeat/Cas9), RNA interference (RNAi), and viral vectors. Finally, we highlighted some candidate genes that are involved in various biological pathways and may contribute to the resistance of chickens against the diseases.

## Genetic roles in host resistance and susceptibility

The MHC gene is widely evaluated in chickens to identify differences in their resistance and susceptibility to certain pathogens and infectious diseases. MHC class I, II, III, and IV molecules are unique and distinct between species, leading to a differential MHC response among individuals (3). Chickens have few MHC genes with different haplotypes involved in the development of resistance against bacterial, viral, and protozoal pathogens. For instance, MHC haplotype B19 is associated with susceptibility, while B2 and B21 are involved in resistance (18). MHC-dependent resistance and susceptibility rely on peptide-binding specificity. For example, chicken-affected cells expressing MHC-I haplotype, which binds to the Rous sarcoma virus src peptide targeted by cytotoxic CD8+ T cells, are resistant to Rous sarcoma virus (19). In susceptible chickens, the MHC haplotype does not bind with viral peptides, and chickens are infested by the virus. For instance, the MHC class I haplotypes do not bind to the antigenic peptides of Marek's disease virus (MDV), resulting in chickens remaining susceptible (19). The chicken MHC haplotype has a regulatory effect on immune cells resistant to the Rous sarcoma virus and exhibit enhanced natural killer cell activity (20). In a recent study, the MHC haplotypes B15 and B21 homozygotes led to the lowest MDV-induced tumorigenesis and lymphoma formation in VALO specific pathogen-free chickens, demonstrating that MHC conferred resistance to oncogenic herpesviruses (21). Notably, the MHC-peptide complexes engaged T cell receptors (TCRs) that recognize antigens on MHC molecules with the cooperation of CD4+ or CD8+ coreceptors and activate T cells (22). Each T cell has a unique TCR that recognizes and binds with the antigenic peptide on the infected cell surface. The antigen peptides are derived from intracellular pathogens, such as viruses and bacteria, and are displayed at the cell surface by MHC for immune clearance (23). Viruses, such as the avian leucosis virus, have six subgroups, with subgroup J causing severe outbreaks in China. The avian leucosis virus subgroup J receptor is a sodium/hydrogen exchanger 1, which is edited on chicken somatic cell lines that are resistant to avian leucosis virus *in vitro* (24). Avian influenza virus replication is facilitated by the acidic leucine-rich nuclear phosphoprotein-32A (ANP32A) protein in chicken and waterfowl. An *in vitro* study revealed that the deletion of a minor region of chicken ANP32A stops the replication of the avian influenza virus (25). Although such studies have increased our understanding of the genetic roles, a functional study of edited ANP32A and sodium/hydrogen exchanger 1 gene has not been performed *in vivo* and edited chicken has hitherto been developed.

**Figure 1:**
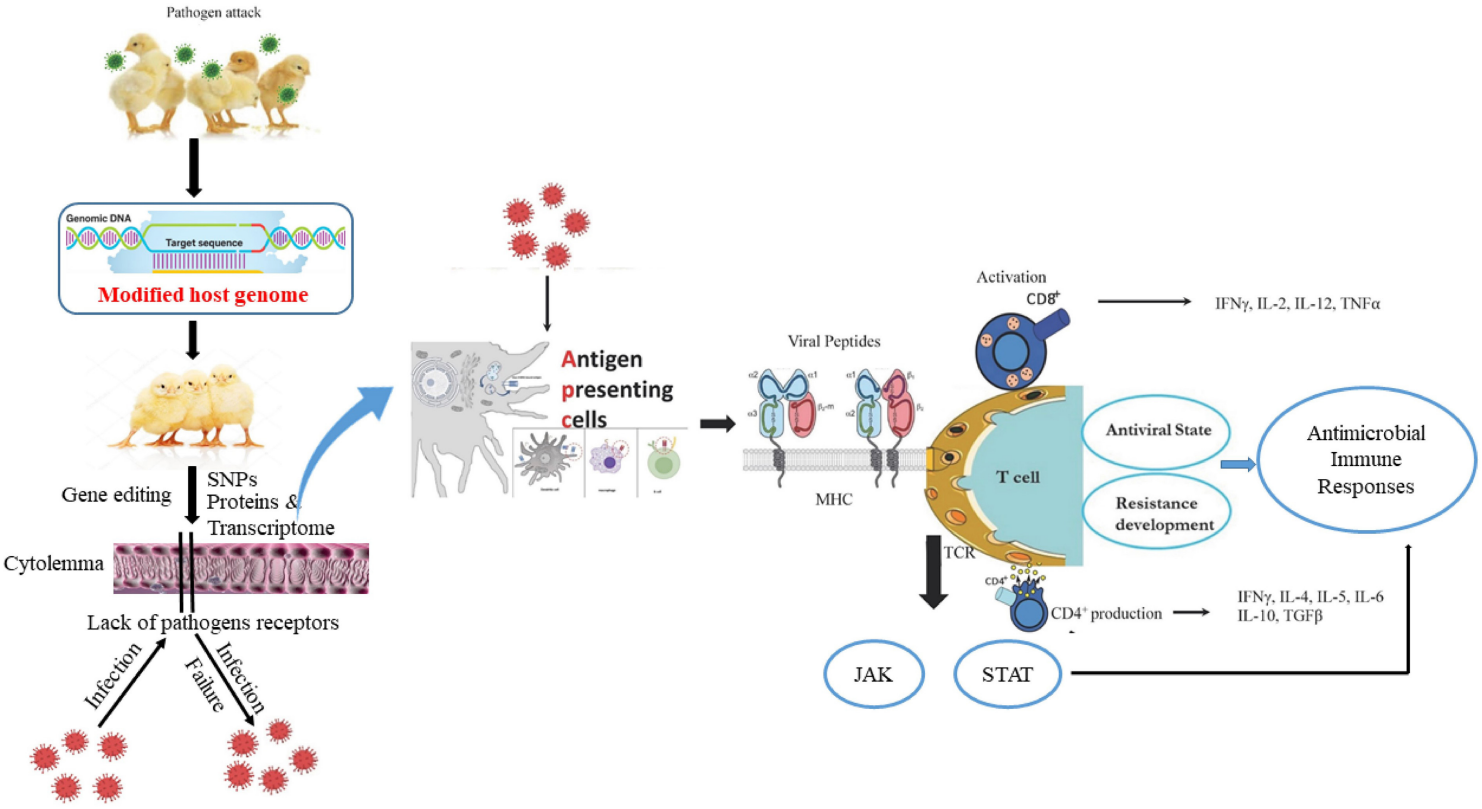
The host edited-genome and active immune responses of chicken during pathogen attack. Usually, chicken antigen presenting cells (dendritic cell, macrophage, B cell) engulf, digest, and present pathogen antigen on the cell surface in conjunction with an MHC molecule. The MHC/peptide complex stimulates the TCR and activates CD4+ and CD8+ cells. Accordingly, cytokines are produced, cell-signaling pathways such as JAK/STAT are activated, and a disease resistance state is developed inside the chicken that counters the pathogen virulence.

Generally, increasing poultry resistance to infectious pathogens *via* gene modification is an ideal approach for the development of transgenic livestock. In particular, resistance to diseases originates from the interplay of numerous genes. For example, the mouse fibroblast cell lines are resistant to influenza virus owing to the autosomal dominant Mx1 allele of the murine Mx gene (26). The introduction of the Mx1 gene in mice lacking the Mx1 allele leads to influenza-resistant mice (27) whereas transfer of the same gene in swine does not result in viral-resistant pigs (28). The overexpression of pathogen anti-receptor proteins blocks viral attachment and penetration, and alters the host receptor genes that prevent viral attachment and enhanced resistance against diseases (29). Transgenic chickens express a recombinant avian leukosis envelope protein, which inhibits the corresponding subgroup of avian leukosis virus attachment (30). Similarly, transgenic sheep express the Maedi-visna virus envelope protein and display resistance *via* the prevention of virus adhesion to host cells (31). Collectively, the observed immune responses of chicken against viral, protozoal, and bacterial agents are pathogen-specific, and are closely linked to expression changes in MHC, Nramp-1, RIP2, MyD88, IFN, interleukin, MX1, TLR4, antibodies, and immune cells that govern antibacterial and antiviral states (Figure 1).

### Genetic resilience and viral pathogens

Viral diseases cause more outbreaks, reduce growth performance and productivity, and cause immunosuppression in poultry (3). Marek's disease, a well-known viral disease, is caused by MDV. Marek's disease is a lymphotropic disease in chickens and the MDV targets all avian species, causing symptoms such as paralysis, loose watery stool, lymphomas, wasting, and immunosuppression. The poultry response to Marek's disease is the activation of MHC molecules and cytokines that give resistance to MDV (22). Other genes that confer resistance to MDV include *GH1, SCA2, IRG1, CD79B, PTPN3, LY6E*, and *SMOC1* (32). Another important virus is influenza, a zoonotic virus that causes avian flu. Genes, such as interferon-inducible transmembrane, a retinoic acid-inducible gene I, and MX1 gene polymorphisms are reported to be associated with susceptibilities to avian influenza in chickens and ducks (33, 34). Newcastle disease virus widely infects chickens. Newcastle Disease is characterized by ruffled feathers of chicken, and respiratory, neurological, hyperthermia, and listlessness complications in affected chickens (35). Potential genes, such as *IFN*α, *IFN-*γ, *DDX-1, MHC-1*, and *IL-6*, were identified in chicken embryos infected with Newcastle disease virus. These important genes have an antiviral function and induce TLR-mediated activation of macrophages and dendritic cells in response to viruses (36). Newcastle disease virus-infected Fayoumis birds were found to have lower expression of EIF2B5, EIF2S3, EIF2B4, and EIF2S3 than Leghorn's infected lines. Such results indicate that different genetic lines display different expression of host translation initiation factor-2 associated genes, which might contribute to their differential resistance to Newcastle disease virus (37). In a study conducted in Ghana, three Ghanaian local chicken ecotype responses to the lentogenic and velogenic strains of Newcastle disease virus assessed. Based on the findings, resistance to Newcastle disease virus was identified to be caused by an individual's chicken genetic makeup and not by the chicken ecotype (38). The genes, MHC-B locus, *LEI0070, ADL0146, LEI0104, ADL0320*, and *ADL0304*, are associated with a direct response of antibody titer against Newcastle disease virus in chickens (39). Wang et al. (40) revealed that the hemoglobin family genes, functional involvement of oxygen transportation and circulation, and cell adhesion molecule signaling pathway play significant roles in disease resistance to AIV infection in chickens. The influenza H5N1 strain was inoculated into genetically resistant and susceptible Ri chicken native to Vietnam. The resistant chicken displayed a group of genes, *MX1, TLR3, STAT1, IRF7, IFN*, and cytokines, which are found in H5N1 strain-resistant chickens (41). Avian Leukosis virus infection is highly receptor-specific and the Leukosis virus subtype A uses specific membrane proteins, such as Tva receptors for binding, CAR1 receptors for avian Leukosis virus subtypes B & D attachment, and SEAR receptors for Avian Leukosis virus subtype E, which is encoded by tumor virus genes (42). These chicken breeds express certain receptors on their cell surface, such as Tva and CAR1, and are susceptible to the corresponding avian Leukosis virus subtype (42, 43). Chickens resistant to infectious bronchitis, Newcastle disease, Marek's disease, coccidiosis, and salmonellosis had high production of IFN-γ, which validated the enhanced production of Th1 and cytotoxic T cells (44). By examining fowl Adenovirus serotype 4 infection, which causes hepatitis hydropericardium syndrome in poultry, Xiang et al. (45) revealed that the expression levels of IL-6, IL-1β, IFN-α, JAK, and STAT were significantly high after viral infection. In summary, during infection, the host induces changes in gene expression that confer transient or long-lasting protection against pathogens. Exploring why, when, which, and how a host reprograms its genome against infectious pathogens is an exciting research topic that can reveal the amplitude of virulence and its genetics.

### Genetic resilience and autoimmune diseases

The TCR exhibits polymorphism that creates high diversity and differences in disease response by T cells (38). The TCR diversity is due to gene rearrangement where different segments, including variable (V), diversity (D), and joining (J) segments of the TCR gene, randomly recombine, and genes for the α, β, γ, and δ chains are formed (46). Although chickens have few V, D, and J genes that limit TCR diversity, TCR heterodimers can be created. For example, the TCR heterodimers of the α and β chains are the αβ T cells distinguished by the V region of the β-chain that causes Vβ1+ (TCR 2) and Vβ2+ (TCR 3) with functional multiplicity (47). TCR defects in chicken are associated with susceptibility to autoimmune diseases. In fact, the TCR defects in scleroderma disease cause low CD4+ cells and non-specific T cell response in chicken (48). Moreover, autoimmune thyroiditis disease is prevented by the depletion of CD4+ cells, highlighting the involvement of the TCR Vβ1 gene (49). Coccidiosis-resistant chicken lines have a high number of CD4+ cells whereas susceptible chickens have a high number of CD8+ cells (50). Moreover, a low number of CD8+ was detected in turkeys infected with Newcastle disease virus, *Pasteurella multocida*, and *Erysipelothrix rhusiopathiae* (50) whereas a high number of CD8+ cells was found in amyloidosis-resistant chickens compared to susceptible chickens (51). Altogether, the amount of CD4+ and CD8+ in resistant or susceptible birds does not align with a particular disease or pathogen in poultry, which might be due to the polymorphism of CD8+ and CD4+. CD4+ cells exhibit resistance toward non-intracellular while CD8+ cells exhibit toward intracellular pathogens that direct differential immune responses against a pathogen (52, 53). In conjunction with cellular immunity, humoral immunity plays a very key role in resistance to diseases. Immunoglobulin genes produce antibodies, and chickens with high antibody production display resistance against microbes, such as *Mycoplasma gallisepticum, Escherichia coli*, Newcastle disease virus, and *Salmonella enteritidis* relative to low antibody producers (54, 55). Chicks that are high antibody producers have numerous CD4+ and type II helper T lymphocytes (Th2), whereas low antibody producers have numerous CD8+ cells and type I helper T lymphocytes (Th1) that improve their resistance against pathogens (56, 57). The Th1 cytokines include IFN-γ, IL-2, and IL-12 whereas the Th2 cytokines include IL-4, IL-5, IL-6, and IL-10, which stimulate cell-mediated and antibody responses, respectively (58). Altogether, variations exist in cellular and humoral immune responses in different chicken breeds, and high expression of cytokines leads to a higher immunocompetence of the host.

### Genetic resilience and bacterial pathogens

Bacterial invasiveness in chickens depends on the species, severity, and virulence of the pathogen. The predominant bacterial pathogens affecting poultry are *Escherichia coli, Campylobacter jejuni, Clostridium perfringens*, Mycoplasma, and *Salmonella spp*. In contrast, *Erysipelothrix rhusiopathiae*, Gallibacterium anatis, *Pasteurella multocida, Riemerella anatipestifer, Avibacterium paragallinarum, Ornitobacterium rhinotracheale*, and *Bordetella avium* are infrequently detected (59, 60). The most devastating bacteria in terms of yield in the poultry industry belong to the genus, *Salmonella*, and include the species, *S. enterica* and *S. bongori*, that easily infect the newly hatched chicks and cause a decline in growth, egg production, and hatchability in chickens (61). To counter salmonellosis, prophylactic measures, such as antibiotics, vaccination, and disease management, are insufficient in poultry flock surveillance (62). Accordingly, the importance of resistant-chickens has increased, and the development of disease-resistant traits through genetic improvement has become more significant. Chicken MHC I, MHC II, Nramp-1, heterophils, IFN γ, and interleukins are involved in Salmonella-specific antibody responses and lead to resistance to salmonellosis (63). In a previous study, heterophils from chicken resistant to *S. enteritidis* had a higher level of cytokine mRNA than heterophils isolated from susceptible chickens (64). The mRNA level of interleukins and IFN γ increased in resistant chicks relative to that in chickens susceptible to salmonellosis (65). IFN γ plays a significant role in the eradication of Salmonella carriers and persistence state (66). The genes, *Nramp-1* and *Nramp-2*, are the macrophage proteins expressed in heterophils and leukocytes that facilitate *S. enteritidis* phagocytosis in resistant chicks (67). Other genes, such as transforming growth factor B4 (*TGFB4*) and *Sal1*, are involved in controlling Salmonella and other bacterial loads in the spleen, and have been linked to increasing genetic resistance against *S. enteritidis* (68). In a recent study, Beijing-You and Cobb chicks were orally challenged with *S. typhimurium*, which revealed robust responses of natural killer-cell-mediated-cytotoxicity, phagosomes, cytokines, MHC, and antibody production in Beijing-You chicken, ultimately indicating the greater resistance of Beijing-You breed to *S. typhimurium* (69). The chicken RIP2 pathway plays a significant role in resistance against avian pathogenic *E. coli* infection. *E. coli* infection promotes RIP2 expression and inhibits cell viability, whereas knockdown of RIP2 restores cell viability and represses the apoptosis of chicken HD11 cells. Nuclear factor I B increases the expression of RIP2, reduces cell viability, and accelerates *E. coli*-induced apoptosis, confirming that RIP2 supported *E. coli* proliferation in chicken cells (70). *Mycoplasma gallisepticum* infects the lungs of chickens and causes chronic respiratory disease. Glycyrrhizic acid is a herb that has anti-inflammatory, anti-microbial, and antioxidant activities and inhibits *M. gallisepticum* infection by suppressing the expression of matrix metalloproteinases through the JNK (c-Jun N-terminal kinase) and p38 pathways and inhibiting the expression of virulence genes of *M. gallisepticum* (71). *Campylobacter jejuni* infections are prevalent in poultry and colonize the intestine of birds. The bird's response to *C. jejuni* is similar to Salmonella infection, and high expression levels of cytokines, T and B cells, and antibodies are detected in *C. jejuni*-resistant birds relative to susceptible birds (72), except quantitative trait loci localization, which is located in different chromosomes (73). Breeder selection of traits that correlate with enhanced resistance against pathogens is highly desirable, and can be determined *via* extensive immunogenetics research. Therefore, screening host genome for disease-resistance genes and pathways in chickens can pave the way for the development of immunocompetent chickens.

### Genetic resilience and protozoans

The next important etiological agents that cause infectious diseases in chicken are protozoal parasites, including *Eimeria tenella, Ascaridia galli*, and *Histomonas meleagridis*. The protozoan, *H. meleagridis*, causes blackhead disease or histomoniasis (74); *E. tenella* causes coccidiosis in chickens (75); and *A. galli* infects chickens and turkeys and causes stunted growth and enteritis (76). Pathogen-specific immune responses occur against parasitic infections in chicken. For instance, the myeloid leukemia factor 2 gene help in resistance to Eimeria (77), and the *IFNG* gene is associated with *Ascaridia* resistance in poultry (78). Moreover, the MHC haplotypes protect the jungle fowl from coccidian (79) and chicken lines from *Ascaridia* infections (80). Other genes, such as TGFβ 2-TGFβ 4, Caspase-1, inhibitor of apoptosis protein1, prosaposin, inducible nitric oxide production, IL-2, immunoglobulin light chain, and tumor necrosis factor-related apoptosis-inducing ligand, have been relatively less explored in protozoan resistance, but can improve the disease resistance traits in poultry.

Yang et al. (81) discovered that butyrate, forskolin, and lactose compounds synergistically increase the expression of multiple host defense peptides, improve the survival of chickens, and reduce the colonization of *Eimeria maxima* and *Clostridium perfringens*. A list of candidate genes in poultry that exhibit important functional activities in animals, but have not been explored for disease resistance in chickens, is provided in Table 1.

**Table 1:** The key genes are involved in the infectious diseases of chickens.

**Gene ID**	**Gene symbol**	**Gene name**	**Access code**	**Study type**	**Function**	**Location**	**Reference**
396241	*TF*	*Transferrin*	NM_205304.2	*In vitro*	Iron-binding glycoprotein and involved in anti-microbial activities, against Marek's disease	*chromosome: 9*	(7, 82)
416928	*IGLL1*	*Immunoglobulin lambda-like polypeptide 1*	NM_001278545.1	*In vivo*	Antibacterial properties against *Streptococcus mutans*	*chromosome: 15*	(83)
100543636	*LOC100543636*	*Ovoinhibitor*	XM_010719004.3	*In vitro*	Antibacterial activities during embryo developments	*chromosome: 15*	(7, 84)
424533	*VTG2*	*Vitellogenin 2*	NM_001031276.2	*In vivo*	Transfer of nutrients for developing embryo and reduce intestinal oxidative stress	*chromosome: 8*	(7, 85)
418974	*VMO1*	*Vitelline membrane outer layer 1*	NM_001167761.2	*In vivo*	Diagnostic marker of ovarian cancer in hen	*chromosome: 1*	(86)
395364	*PIT54*	*PIT54 protein*	NM_207180.2	*In vivo*	Hemoglobin-binding protein of plasma in chicken which has antioxidant activity	*chromosome: 31*	(87)
420897	*OVALY*	*Ovalbumin-related protein Y*	NM_001031001.1	*In vitro and In vivo*	Ovalbumin has antioxidant and radical scavenging activities	*chromosome: 2*	(88, 89)
396393	*EX-FABP*	*Extracellular fatty acid-binding protein*	NM_205422.2	*In vitro*	Function as an antibacterial siderophore binding lipocalin	*chromosome: 17*	(90)
395722	*CLU*	*Clusterin*	NM_001396177.1	*In vivo*	Serve as a marker for follicular atresia and involve in developmental phases of follicles	*chromosome: 3*	(91)
396384	*IRF1*	*Interferon regulatory factor 1*	NM_205415.2	*In vitro*	Inhibits the replication of avian influenza virus and Newcastle disease virus	*chromosome: 13*	(92)
769014	*TLR2*	*Toll like receptor 2*	NM_001161650.3	*In vivo*	Immunity and resistance to bacterial infection	*chromosome: 4*	(93)
395764	*CASP1*	*Caspase 1*	NM_001161650.3	*In vitro and In vivo*	Involved in apoptosis, necrosis, mitophagy, and autophagy	*chromosome: 19*	(94–96)
418300	*ZYX*	*Zyxin*	NM_001004386.2	*In vivo*	Zyxin is a candidate gene potentially associated with increased resistance to experimental avian coccidiosis.	*chromosome: 1*	(97)
396260	*AVD*	*Avidin*	NM_205320.2	*In vitro*	Antimicrobial activity	*chromosome: Z*	(98, 99)
418812	*ACOD1*	*Aconitate decarboxylase 1*	NM_001030821.2	*In vivo*	Antimicrobial activity	*chromosome: 1*	(100)
374125	*LITAF*	*Lipopolysaccharide induced TNF factor*	NM_204267.2	*In vitro and In vivo*	Initiates the activation of caspases and kinase protein signaling of the cell death pathway and has antimicrobial activity	*chromosome: 14*	(101)
395283	*TRAIL-LIKE*	*TNF-related apoptosis inducing ligand-like*	XM_015278184.4	*In vitro and In vivo*	It declines the autoimmune response by suppressing cell cycle progression.	*chromosome: 4*	(102)
420963	*PTPN3*	*Protein tyrosine phosphatase, non-receptor type 3*	XM_419047.8	*In vivo*	Involved in immune suppression disease.	*chromosome: 2*	(103)
378897	*THY1*	*Thy-1 cell surface antigen*	NM_204381.3	*In vitro*	Involved in chicken Marek disease.	*chromosome: 24*	(104)
768688	*SMOC1*	*SPARC related modular calcium binding 1*	XM_015287582.4	*In vivo*	Enhanced endothelial cell proliferation and angiogenesis.	*chromosome: 5*	(105)
422993	*LOC422993*	*Interferon-induced transmembrane protein 3-like*	NM_001350059.2	*In vivo*	Highly expressed in response to avian Tembusu virus infection	*chromosome: 5*	(106)

## SNP-dependent resistance and susceptibility in chickens

SNP is the nucleotide sequence variation that occurs at a single position in DNA fragments and is extensively used as a molecular marker in genetic studies. The roles of SNPs are largely associated with production traits in chicken. SNPs have been detected in follicle-stimulating hormone, prolactin receptor, dopamine receptor 2, low-density lipoprotein receptor-related protein, and luteinizing hormone receptors, with characteristic changes in duck and chicken (107, 108). For instance, the follicle-stimulating hormone regulates reproductive activities in birds, and the SNP detected in the follicle-stimulating hormone is linked to the reproductive traits of chickens (109). Two key SNPs, A227G and C320T, were identified in the Muscovy duck follicle-stimulating hormone gene that improve egg production traits (110). Ye et al. (111) revealed two SNPs in the insulin-like growth factor 2 gene and 11 SNPs in dopamine receptor 2 that are linked with egg-laying traits (111). In a recent study, the polymorphism of the DMA gene, a member of the non-classical MHC class II gene, was associated with disease resistance traits in broiler chickens. Four SNPs linked to seven haplotype formations were found, with haplotypes 1 and 5 associated with high immunoglobulin yolk concentration and ND antibody level, respectively (112). The SNPs detected in the carboxypeptidase Q and leucine-rich repeat transmembrane neuronal 4 gene regions resulted in a decrease in pulmonary hypertension syndrome and greater innate ascites resistance in chicken offspring (113). Mountford et al. (2) correlated SNPs with resistance and susceptibility to MDV, infectious bursal disease virus, avian influenza virus, and infectious bronchitis virus. These researchers detected 10 SNPs that were involved in the resistance to MDV and 8 SNPs associated with the susceptibility to infectious bursal disease virus. Recently, IL10Rβ SNP resulted in an R318K amino acid substitution that was involved in the enhanced regulation of the type III interferon pathway that reduced bursal damage in infectious bursal disease virus-infected birds (114). A previous study revealed the same SNP involvement in increased susceptibility to MDV (115). Thus, IFN response can vary for viruses owing to viral mutagenicity and strain diversity. As a result, viruses can block the IFN responses. Nramp-1, Sal-1, and Tnc are the genes involved in resistance to *Mycobacterium, Salmonella*, and *Leishmania* infections (116). In chicken, Nramp-1 polymorphism is correlated with susceptibility to salmonellosis. Frequent sequence variations were detected in this gene that conferred resistance differences in chicken (4, 117). MyD88 polymorphism is associated with *S. pullorum* susceptibility in chickens and has a favorable effect on vulnerability to *S. pullorum* infection (118). These recently identified SNPs are associated with disease-resistance genotypes that can help in the identification of new genes and their roles in eradicating infectious diseases.

## Non-coding RNA resistance in chicken

Non-coding RNAs are biological molecules involved in epigenetic regulation and disease resistance (119). There are different classes of non-coding RNAs, such as circular RNAs, small interfering RNAs, long non-coding RNAs (LncRNAs), microRNAs, and transfer RNAs, that play important roles in avian immunity and cell development. Among these classes of RNAs, LncRNAs, circular RNAs, and microRNAs are called regulatory RNAs that mediate gene expression in different hosts (120). LncRNAs are longer than 200 nucleotides and are known as signaling molecules that interact with mRNA, miRNA, DNA, and proteins, thereby regulating various processes, such as apoptosis, tumor cell invasion, RNA transcription, and host resistance to pathogen infection (121, 122). Based on recent studies, lncRNAs regulate vitamins A and D during bacterial and fungal infections and activate immune responses during chicken leukemia virus infection (123). Specialized lncRNAs that reduce the production of inflammatory cytokines, such as IL-6, IL-8, and TNF-α, were identified in response to *E. tenella* infection in chickens (124). An important ERL lncRNA acts as an antisense transcript of MDV carcinogen, is expressed during the lytic and lysogenic phases of viral infection, and inhibits the expression of MDV miRNAs (125, 126). The lncRNA, GLAMD3, cis-regulates the gga-miR-223 expression that targets IGF1R (insulin-like growth factor 1 receptor), which regulates Marek's disease lymphoma (127). Another important lncRNA, linc-stab1, regulates the Marek's disease resistance gene, SATB1, which is also involved in cell-mediated immunity for termination of MDV-infected cells (128).

Several studies revealed the roles of circRNAs in avian leukosis virus infection. Furthermore, differentially-expressed circRNAs were detected in infected organs. circRNAs are involved in T and B-cell activation (129), and Jak-STAT pathway regulation (130). In contrast to lncRNAs and circRNAs, the expression profile and functional mechanism of miRNAs are well-characterized in disease resistance in chickens. In fact, differentially expressed miRNAs have a significant effect on oncogenicity (131); the regulation of MAPK, JaK/STAT, and Wnt pathways (132); and suppression of chronic myeloid leukemia caused by avian leukosis virus in chicken (133). In conclusion, non-coding RNAs regulate disease resistance traits, interact with host and pathogen genes, and help to control infectious diseases.

## Modern technology and development of disease-resistant chicken

Gene-editing techniques, such as zinc-finger nucleases (ZFNs), transcription activator-like effector nucleases (TALENs), pronuclear injection, sperm-mediated gene transfer, somatic cell nuclear transfer, recombinases, transposons, viral vectors, and CRISPR/Cas9 systems, are novel molecular tools that are efficiently used in mice, cattle, sheep, and goat. For instance, transgenic mice, rabbits, pigs, and sheep were engineered by microinjection of the target DNA into the fertilized embryo (134, 135); lentiviral vectors and embryonic stem cells were used to produce germline transgenic birds (136, 137); and successful knock-out in chickens were achieved by homologous recombination in primordial germ cells (138). In zinc-finger nucleases and transcription activator-like effector nucleases techniques, the proteins bind to the target DNA sequence for modification, whereas the CRISPR requires a guide RNA to recognize the target DNA fragments. Further, the endonuclease enzyme performs a target-specific cut (139). Since the introduction of the CRISPR/Cas9 system in genome editing, substantial progress has been made in the use of the CRISPR/Cas9 technology in chickens. A CRISPR/Cas9-mediated chicken was engineered in 2015 (139) and ovomucoid gene-targeted chickens and knocked-in of human interferon beta into the chicken ovalbumin gene were edited successfully (140, 141). The emerging viral strains of avian leukosis and MDV are highly pathogenic. Further, existing vaccines and antiviral drugs are becoming less effective. Thus, novel antiviral strategies are needed. For instance, through CRISPR/Cas9, the avian leukosis virus subgroup J receptor sodium/hydrogen exchanger type 1 is mutated, which protects the chicken line from avian leukosis virus subgroup J. Subgroup J prototype strain replication is also impaired in mutated birds (142). Resistance was found to develop in chicken cells against avian leukosis virus subgroup J by creating tryptophan mutations at position 38 (143). In another study, genetic resistance to avian leukosis virus subgroups A, C, J was induced by creating frame-shift mutations in *tva* (tumor virus locus A gene), *tvc*, and *tvj* genes (144). Koslová et al. (143) and Hellmich et al. (144) produced ALV-J-resistant chicken lines *via* precise gene editing of chicken sodium/hydrogen exchanger 1. A recent study revealed that transgenic chickens constitutively express Cas9 and guide RNAs specific to the immediate early infected-cell polypeptide-4 (gICP4) of MDV upon challenge with MDV, and exhibit reduced replication compared to wild-type chickens (145). These examples highlight the use of the CRISPR/Cas9 system to edit genes of interest and engineer chicken flocks that exhibit resistance characteristics to viral infection (146). Lately, CRISPR/Cas9 has been used to develop transgenic animals. Accordingly, transgenic animals are generated *via* the targeted placement of *Streptococcus pyogenes* Cas9 at the ROSA26 locus and endogenous pseudo attP site in pigs and chickens, respectively. Transgenic chickens and pigs constitutively express Cas9. Cas9 was confirmed in pigs and chicken for different target genes in many cell types with the *S. pyogenes* Cas9 platform for *in vitro* and *in vivo* genome editing in livestock species (147). Similarly, different computational and bioinformatics approaches can be used to design synthetic RNA duplexes that would target the mRNA sites of viral, bacterial, and protozoal pathogens. For example, synthetic RNA duplexes that target specific domains of viral genes can inhibit viral replication (148). Techniques, such as RNA interference technology, have strong applications in the development of transgenic poultry that is resistant to microbial infections. RNA interference is the method of choice, where RNA molecules inhibit gene expression by targeting specific mRNA. Similarly, a lentiviral vector containing influenza-specific RNA hairpin rendered the cells refractory to viral infection and inhibited influenza virus replication in mice (149). These results provide evidence and scope for the development of pathogen-resistant poultry flocks *via* the transgenic expression of gene-specific RNA. In an earlier study, a recombinant plasmid with synthetic RNA duplex gene was constructed and transferred into Madin-Darby Canine Kidney cells. The study revealed that the transfected cell lines were resistant to the avian influenza virus (150). This landmark experiment provided the breakthrough for transgenic chicken development and resistance to influenza virus. RNA-Sequencing is another advanced technique that reveals the poultry genome responses to different stresses and diseases. The development of a disease-resistant chick through traditional breeding is a difficult and labor-intensive task, while the use of gene-editing technology and production traits is time-saving and profitable (151). With the development of next-generation sequencing technology, interest in whole-genome sequencing as an alternative to SNP chips for genotyping has increased as it allows the capture of a wide range of variations. For instance, a genome-wide association study and quantitative trait loci mapping identified candidate genes for egg production in ducks (152). These tools would help in the editing of the chicken genome and fulfill the dire need for disease-resistance breeds in poultry.

## Applications of chicken-genomics in biomedical research

Chickens are widely used in developmental research owing to their easy rearing, fecundity, growth rates, and genetic variations, thereby advancing the field of biomedical research. The chicken model has been used to evaluate cancer metastasis, test chemotherapy agents, tissue morphogenesis, and angiogenesis, and perform toxicology studies. The egg is an important source of protein and contains phosvitin, which protects against oxidative stress-induced DNA damage in human leukocytes (153), and ovotransferrin, which is used as growth inhibitor for cancer cell lines (154). Avian-derived cell lines are used for viral culture and are helpful in vaccine and recombinant protein production (155). Chicken has also been used as a xenotransplantation model for human stem cells (156), human multiple myeloma xenograft (157), and the production of human antibodies (158). The Omni Chicken by Ligand Pharmaceuticals Inc. is a worldwide unique platform used to produce human monoclonal antibodies from chickens (159). Oishi et al. (141) integrated human interferon beta (hIFN-β) into the chicken ovalbumin locus and produced hIFN-β in egg white. Notably, antibodies produced from humanized chickens and antibodies produced in chicken eggs represent significant industrial applications. Accordingly, chicken is an attractive developmental model for biomedical research.

## Conclusion

This review summarized the disease-resistance genes in poultry and provided an outlook of advanced technologies that can be used to engineer disease-resistance characteristics in poultry. The poultry industry is one of the fastest growing sectors of livestock for meat and egg production; however, this industry is threatened by different pathogens, which lead to substantial economic losses. Vaccination, antibiotics, culling, and disease management techniques are frequently employed in flocks to control disease outbreak; however, the success rate is nominal. Genetic resistance is a promising alternative method to augment prophylactic measures. Genetic resistance can be acquired through genetic breeding and genetic modification. Breeding chickens with disease-resistant strains can increase flock resistance; however, the genome modification process can underpin a characteristic of interest and assimilate into offspring to improve immune responses. Currently, genome editing technologies are driving desirable phenotypic traits, as genetic modifications are meeting enhanced production goals in the poultry industry, and engineering elite chicken for breeders. Further studies are required to effectively determine the roles of candidate genes in generating an ideal disease-resistant chicken.

## Future prospective

Next-generation sequencing of chicken-genome and pathogens helps in the understanding of host-pathogen interactions, natural variations, and the discovery of new QTLs that may be associated with disease-resistance and susceptibility traits in poultry. The use of lentiviral vectors is very efficient for gene delivery in animals and poultry compared to homologous recombination of embryonic and somatic cells. Other alternatives for embryonic stem cells include RNAi and ZFNs technologies, which may be used for gene targeting and disruption in animals. The amplified genomic information of poultry and the advent of more sophisticated transgenic tools would result in resistance against pathogens. By investigating the genomics of chickens, new genes with divergent characteristics may lead to enhanced chicken yield. The use of other bird species with similar and unique characteristics will also advance avian research.

## Author contributions

HG, IK, and YL contributed to conceptualization. GH contributed to the methodology. IK and SR contributed to software. YL and HG contributed to validation and original draft. NK and SR contributed to investigation. YL contributed to resources and funding acquisition. GH, IK, HW, and NK contributed to the final draft and editing. All authors contributed to the article and approved the submitted version.

## Funding

This work was funded by Industry-University-Research Project of Fuyang Normal University (HX2021027000 and HX2022048000).

## Conflict of interest

The authors declare that the research was conducted in the absence of any commercial or financial relationships that could be construed as a potential conflict of interest.

## Publisher's note

All claims expressed in this article are solely those of the authors and do not necessarily represent those of their affiliated organizations, or those of the publisher, the editors and the reviewers. Any product that may be evaluated in this article, or claim that may be made by its manufacturer, is not guaranteed or endorsed by the publisher.

## References

[1] Gogoi A Das B Chabukdhara P Phookan A Phangchopi DJAR. Livestock breeding for disease resistance: a perspective review. Agric Rev. (2022) 43:116–21. 10.18805/ag.R-2169

[2] Mountford J Gheyas A Vervelde L Smith JJAG. Genetic variation in chicken interferon signalling pathway genes in research lines showing differential viral resistance. Anim Genet. (2022) 53:640–56. 10.1111/age.13233 35739459 PMC9544680

[3] Zekarias B Ter Huurne AA Landman WJ Rebel JM Pol JM Gruys E. Immunological basis of differences in disease resistance in the chicken. Vet Res. (2002) 33:109–25. 10.1051/vetres:2002001 11944802

[4] Dar MA Mumtaz PT Bhat SA Nabi M Taban Q Shah RA. Genetics of disease resistance in chicken. Appl Genet Genomics Poult Sci. (2018) 168–74. 10.5772/intechopen.77088

[5] Alberts B Johnson A Lewis J Raff M Roberts K Walter P. T cells and MHC proteins. In: Molecular Biology of the Cell. 4th edition. New York, NY: Garland Science (2002).

[6] Kinome M. Metabolism when stimulated early in life with CpG. Poult Sci. (2022) 101:101775. 10.1016/j.psj.2022.101775 35299064 PMC8927827

[7] Gul H Chen X Geng ZJA. Comparative yolk proteomic analysis of fertilized low and high cholesterol eggs during embryonic development. Animals. (2021) 11:744. 10.3390/ani11030744 33803097 PMC8035655

[8] Gul H Geng Z Habib G Hayat A Rehman MU Khan IJTAH. Effect of ellagic acid and mesocarp extract of *Punica granatum* on productive and reproductive performances of laying hens. Trop Anim Health Prod. (2022) 54:228. 10.1007/s11250-022-03222-7 35809139

[9] Stear M Bairden K Bishop S Buitkamp J Duncan J Gettinby G. The genetic basis of resistance to Ostertagia circumcincta inlambs. Vet J. (1997) 154:111–9. 10.1016/S1090-0233(97)80049-4 9308398

[10] Cole R. Studies on genetic resistance to marek's disease. Avian Dis. (1968) 12:9–28. 10.2307/1588081 5643702

[11] Heringstad B Klemetsdal G Ruane JJLPS. Selection for mastitis resistance in dairy cattle: a review with focus on the situation in the Nordic countries. Livest Prod Sci. (2000) 64:95–106. 10.1016/S0301-6226(99)00128-1

[12] Wilkie B Mallard BJ. Genetic aspects of health and disease resistance in pigs. In: Breeding for Disease Resistance in Farm Animals 2. (2000). p. 379–96.

[13] Lyall J Irvine RM Sherman A McKinley TJ Núñez A Purdie A. Suppression of avian influenza transmission in genetically modified chickens. Science. (2011) 331:223–6. 10.1126/science.1198020 21233391

[14] Yu M Muteti C Ogugo M Ritchie WA Raper J Kemp S. Cloning of the African indigenous cattle breed Kenyan Boran. Anim Genet. (2016) 47:510. 10.1111/age.12441 27109292 PMC5074306

[15] Whitworth KM Rowland RR Ewen CL Trible BR Kerrigan MA Cino-Ozuna AG. Gene-edited pigs are protected from porcine reproductive and respiratory syndrome virus. Nat Biotechnol. (2016) 34:20–2. 10.1038/nbt.3434 26641533

[16] Kalds P Zhou S Cai B Liu J Wang Y Petersen B. Sheep and goat genome engineering: from random transgenesis to the crispr era. Front Genet. (2019) 10:750. 10.3389/fgene.2019.00750 31552084 PMC6735269

[17] Golding MC Long CR Carmell MA Hannon GJ Westhusin M. Suppression of prion protein in livestock by Rna interference. Proc Natl Acad Sci U S A. (2006) 103:5285–90. 10.1073/pnas.0600813103 16567624 PMC1459347

[18] Plachy J Pink JR Hala K. Biology of the chicken MHC (B complex). Crit Rev Immunol. (1992) 12:47–79. 1358107

[19] Kaufman J Venugopal K. The importance of MHC for Rous sarcoma virus and marek's disease virus—some payne-ful considerations. Avian Pathol. (1998) 27:S82–7. 10.1080/03079459808419297

[20] Lopez-Botet M Llano M Navarro F Bellon T. NK cell recognition of non-classical HLA class I molecules. Semin Immuno. (2000) 12:109–19. 10.1006/smim.2000.0213 10764619

[21] Bertzbach LD Tregaskes CA Martin RJ Deumer U-S Huynh L Kheimar AM. The diverse major histocompatibility complex haplotypes of a common commercial chicken line and their effect on marek's disease virus pathogenesis and tumorigenesis. Front Immunol. (2022) 13:908305. 10.3389/fimmu.2022.908305 35693787 PMC9186122

[22] Boodhoo N Gurung A Sharif S Behboudi S. Marek's disease in chickens: a review with focus on immunology. Vet Res. (2016) 47:1–19. 10.1186/s13567-016-0404-3 27894330 PMC5127044

[23] Dong K Chang S Xie Q Black-Pyrkosz A Zhang H. Comparative transcriptomics of genetically divergent lines of chickens in response to marek's disease virus challenge at cytolytic phase. PLoS ONE. (2017) 12:e0178923. 10.1371/journal.pone.0178923 28591220 PMC5462384

[24] Lee HJ Lee KY Jung KM Park KJ Lee KO Suh J-Y. Precise gene editing of chicken Na+/H+ exchange type 1 (Chnhe1) confers resistance to avian leukosis virus subgroup J (Alv-J). Dev Comp Immunol. (2017) 77:340–9. 10.1016/j.dci.2017.09.006 28899753

[25] Kolaczkowski B Thornton J. Performance of maximum parsimony and likelihood phylogenetics when evolution is heterogeneous. Nature. (2004) 431:980–4. 10.1038/nature02917 15496922

[26] Staeheli P Haller O Boll W Lindenmann J Weissmann CJC. Mx protein: constitutive expression in 3t3 cells transformed with cloned Mx Cdna confers selective resistance to influenza virus. Cell. (1986) 44:147–58. 10.1016/0092-8674(86)90493-9 3000619

[27] Arnheiter H Skuntz S Noteborn M Chang S Meier EJC. Transgenic mice with intracellular immunity to influenza virus. Cell. (1990) 62:51–61. 10.1016/0092-8674(90)90239-B 2194673

[28] Müller M Brenig B Winnacker E-L Brem GJG. Transgenic pigs carrying Cdna copies encoding the murine Mx1 protein which confers resistance to influenza virus infection. Gene. (1992) 121:263–70. 10.1016/0378-1119(92)90130-H 1446823

[29] Gavora JS. Progress and prospects in resistance to disease. Proc 6th World Congr Genet Appl Livest Prod, Armidale, NSW. Ottawa, ON: Centre for Food and Animal Research Agriculture and Agri-Food Canada. (1998).

[30] Salter D Crittenden LJT Genetics A. Artificial insertion of a dominant gene for resistance to avian leukosis virus into the germ line of the chicken. Theor Appl Genet. (1989) 77:457–61. 10.1007/BF00274263 24232709

[31] Clements J Wall R Narayan O Hauer D Schoborg R Sheffer D. Development of transgenic sheep that express the visna virus envelope gene. Virology. (1994) 200:370–80. 10.1006/viro.1994.1201 8178428

[32] Smith J Lipkin E Soller M Fulton JE Burt DW. Mapping Qtl associated with resistance to avian oncogenic Marek's disease virus (Mdv) reveals major candidate genes and variants. Genes. (2020) 11:1019. 10.3390/genes11091019 32872585 PMC7564597

[33] Sironi L Williams JL Moreno-Martin AM Ramelli P Stella A Jianlin H. Susceptibility of different chicken lines to H7n1 highly pathogenic avian influenza virus and the role of Mx gene polymorphism coding amino acid position 631. Virology. (2008) 380:152–6. 10.1016/j.virol.2008.07.022 18723201

[34] Ichikawa K Motoe Y Ezaki R Matsuzaki M Horiuchi H. Knock-in of the duck retinoic acid-inducible gene I (rig-I) into the Mx gene in Df-1 cells enables both stable and immune response-dependent rig-I expression. Biochem Biophys Rep. (2021) 27:101084. 10.1016/j.bbrep.2021.101084 34381879 PMC8332658

[35] Ezema WS Eze DC Shoyinka SVO Okoye JOA. Atrophy of the lymphoid organs and suppression of antibody response caused by velogenic newcastle disease virus infection in chickens. Trop Anim Health Prod. (2016) 48:1703–9. 10.1007/s11250-016-1147-x 27645826

[36] Zhang J Kaiser MG Deist MS Gallardo RA Bunn DA Kelly TR. Transcriptome analysis in spleen reveals differential regulation of response to newcastle disease virus in two chicken lines. Sci Rep. (2018) 8:1–13. 10.1038/s41598-018-19754-8 29352240 PMC5775430

[37] Del Vesco AP Kaiser MG Monson MS Zhou H Lamont SJ. Genetic responses of inbred chicken lines illustrate importance of EIF2 family and immune-related genes in resistance to newcastle disease virus. Sci Rep. (2020) 10:1–9. 10.1038/s41598-020-63074-9 32273535 PMC7145804

[38] Tudeka CK Aning GK Naazie A Botchway PK Amuzu-Aweh EN Agbenyegah GK. Response of three local chicken ecotypes of Ghana to lentogenic and velogenic newcastle disease virus challenge. Trop Anim Health Prod. (2022) 54:1–12. 10.1007/s11250-022-03124-8 35266056

[39] Touko H Keambou T Han J Bembide C Cho C Skilton RA. The major histocompatibility complex B (MHC-B) and QTL microsatellite alleles of favorable effect on antibody response against the Newcastle disease. Int J Genet Res. (2013) 1:1–8.

[40] Wang Y Lupiani B Reddy S Lamont S Zhou H. RNA-Seq analysis revealed novel genes and signaling pathway associated with disease resistance to avian influenza virus infection in chickens. Poult Sci. (2014) 93:485–93. 10.3382/ps.2013-03557 24570473

[41] Lee J Hong Y Vu TH Lee S Heo J Truong AD. Influenza a pathway analysis of highly pathogenic avian influenza virus (H5n1) infection in genetically disparate RI chicken lines. Vet Immunol Immunopathol. (2022) 246:110404. 10.1016/j.vetimm.2022.110404 35231674

[42] Adkins HB Brojatsch Young JA. Identification and characterization of a shared Tnfr-related receptor for subgroup B, D, and E avian leukosis viruses reveal cysteine residues required specifically for subgroup e viral entry. J Virol. (2000) 74:3572–8. 10.1128/JVI.74.8.3572-3578.2000 10729132 PMC111866

[43] Prikryl D Plachý J Kučerová D Koslová A Reinišová M Šenigl F. The novel avian leukosis virus subgroup K shares its cellular receptor with subgroup A. J Virol. (2019) 93:e00580–19. 10.1128/JVI.00580-19 31217247 PMC6694804

[44] Schat KA Skinner MA. Avian immunosuppressive diseases and immune evasion. Avian Immunology, 3rd Edition. Boston, MA: Academic Press (2022). p. 387–417. 10.1016/B978-0-12-818708-1.00018-X

[45] Xiang S Huang R He Q Xu L Wang C Wang Q. Arginine regulates inflammation response-induced by fowl adenovirus serotype 4 Via Jak2/Stat3 pathway. BMC Vet Res. (2022) 18:1–10. 10.1186/s12917-022-03282-9 35590365 PMC9118595

[46] Davis MM Bjorkman PJ. T-Cell antigen receptor genes and T-cell recognition. Nature. (1988) 334:395–402. 10.1038/334395a0 3043226

[47] Lahti J Chen C Tjoelker L Pickel J Schat K Calnek B. Two distinct alpha beta T-cell lineages can be distinguished by the differential usage of T-cell receptor V beta gene segments. Proc Nat Acad Sci. (1991) 88:10956–60. 10.1073/pnas.88.23.10956 1720556 PMC53051

[48] Wilson TJ Van de Water J Mohr FC Boyd RL Ansari A Wick G. Avian scleroderma: evidence for qualitative and quantitative T cell defects. J Autoimmun. (1992) 5:261–76. 10.1016/0896-8411(92)90142-D 1388634

[49] Cihak J Hoffmann-Fezer G Wasl M Merkle H Kaspers B Hála K. Prevention of spontaneous autoimmune thyroiditis in the obese strain (Os) chickens by treatment with a monoclonal anti-Cd4 antibody. J Vet Med Series A. (1996) 43:211–6. 10.1111/j.1439-0442.1996.tb00446.x 8767730

[50] Vermeulen AN. Avian coccidiosis: a disturbed host-parasite relationship to be restored. Host-Parasite Interact. (2004) 211–41. 10.4324/9780203487709-10 15446451

[51] Zekarias B Landman WJ Tooten PC Gruys E. Leukocyte responses in two breeds of layer chicken that differ in susceptibility to induced amyloid arthropathy. Vet Immunol Immunopathol. (2000) 77:55–69. 10.1016/S0165-2427(00)00233-6 11068066

[52] Luhtala M Tregaskes CA Young JR Vainio O. Polymorphism of chicken Cd8-alpha, but not Cd8-beta. Immunogenetics. (1997) 46:396–401. 10.1007/s002510050293 9271629

[53] Davison T. The immunologists' debt to the chicken. Br Poult Sci. (2003) 44:6–21. 10.1080/0007166031000085364 12737220

[54] Wu Z Ding L Bao J Liu Y Zhang Q Wang J. Co-infection of mycoplasma gallisepticum and escherichia coli triggers inflammatory injury involving the Il-17 signaling pathway. Front Microbiol. (2019) 10:2615. 10.3389/fmicb.2019.02615 31803158 PMC6872679

[55] Gross W Siegel P Hall R Domermuth C DuBoise R. Production and persistence of antibodies in chickens to sheep erythrocytes: 2. Resistance to Infectious Diseases. Poult Sci. (1980) 59:205–10. 10.3382/ps.0590205 6997852

[56] Parmentier HK Kreukniet MB Goeree B Davison TF Jeurissen SH Harmsen EG. Differences in distribution of lymphocyte antigens in chicken lines divergently selected for antibody responses to sheep red blood cells. Vet Immunol Immunopathol. (1995) 48:155–68. 10.1016/0165-2427(94)05411-K 8533310

[57] Parmentier H Abuzeid SY Reilingh GDV Nieuwland M Graat E. Immune responses and resistance to eimeria acervulina of chickens divergently selected for antibody responses to sheep red blood cells. Poult Sci. (2001) 80:894–900. 10.1093/ps/80.7.894 11469651

[58] Infante-Duarte C Kamradt T. Th1/Th2 Balance in Infection. Springer Semin Immunopathol. (1999) 21:317–38. 10.1007/BF00812260 10666776

[59] Skånseng B Kaldhusdal M Rudi K. Comparison of chicken gut colonisation by the pathogens campylobacter Jejuni and clostridium perfringens by real-time quantitative PCR. Mol Cell Probes. (2006) 20:269–79. 10.1016/j.mcp.2006.02.001 16644183

[60] Saif Y. Diseases of Poultry. Ames, IA: John Wiley and Sons (2009).

[61] Andino A Hanning I. Salmonella enterica: survival, colonization, and virulence differences among serovars. ScientificWorldJournal. (2015) 2015:520179. 10.1155/2015/520179 25664339 PMC4310208

[62] Calenge F Kaiser P Vignal A Beaumont C. Genetic control of resistance to salmonellosis and to salmonella carrier-state in fowl: a review. Genet Sel Evol. (2010) 42:1–11. 10.1186/1297-9686-42-11 20429884 PMC2873309

[63] Tohidi R Javanmard A Idris I. Immunogenetics applied to control salmonellosis in chicken: a review. J Appl Anim Res. (2018) 46:331–9. 10.1080/09712119.2017.1301256

[64] Ferro PJ Swaggerty CL Kaiser P Pevzner IY Kogut MH. Heterophils isolated from chickens resistant to extra-intestinal salmonella enteritidis infection express higher levels of pro-inflammatory cytokine mRNA following infection than heterophils from susceptible chickens. Epidemiol Infect. (2004) 132:1029–37. 10.1017/S0950268804002687 15635959 PMC2870193

[65] Swaggerty CL Kogut MH Ferro PJ Rothwell L Pevzner IY Kaiser P. Differential cytokine mRNA expression in heterophils isolated from salmonella-resistant and-susceptible chickens. Immunology. (2004) 113:139–48. 10.1111/j.1365-2567.2004.01939.x 15312145 PMC1782542

[66] Foster N Tang Y Berchieri A Geng S Jiao X Barrow P. Revisiting persistent salmonella infection and the carrier state: what do we know? Pathogens. (2021) 10:1299. 10.3390/pathogens10101299 34684248 PMC8537056

[67] Redmond SB Chuammitri P Andreasen CB Palić D Lamont SJ. Genetic control of chicken heterophil function in advanced intercross lines: associations with novel and with known salmonella resistance loci and a likely mechanism for cell death in extracellular trap production. Immunogenetics. (2011) 63:449–58. 10.1007/s00251-011-0523-y 21455609 PMC3111730

[68] Fife M Salmon N Hocking P Kaiser P. Fine mapping of the chicken salmonellosis resistance locus (Sal1). Anim Genet. (2009) 40:871–7. 10.1111/j.1365-2052.2009.01930.x 20597881

[69] Elsharkawy MS Wang H Ding J Madkour M Wang Q Zhang Q. Transcriptomic analysis of the spleen of different chicken breeds revealed the differential resistance of salmonella typhimurium. Genes. (2022) 13:811. 10.3390/genes13050811 35627196 PMC9142047

[70] Sun H Li N Tan J Li H Zhang J Qu L. Transcriptional regulation of RIP2 gene by NFIB is associated with cellular immune and inflammatory response to APEC infection. Int J Mol Sci. (2022) 23:3814. 10.3390/ijms23073814 35409172 PMC8998712

[71] Wang Y Wang L Luo R Sun Y Zou M Wang T. Glycyrrhizic acid against mycoplasma gallisepticum-induced inflammation and apoptosis through suppressing the MAPK pathway in chickens. J Agric Food Chem. (2022) 70:1996–2009. 10.1021/acs.jafc.1c07848 35128924

[72] Connell S Meade KG Allan B Lloyd AT Kenny E Cormican P. Avian resistance to campylobacter jejuni colonization is associated with an intestinal immunogene expression signature identified by mRNA sequencing. PLos ONE. (2012) 7:e40409. 10.1371/journal.pone.0040409 22870198 PMC3411578

[73] Psifidi A Fife M Howell J Matika O Van Diemen P Kuo R. The genomic architecture of resistance to campylobacter jejuni intestinal colonisation in chickens. BMC Genomics. (2016) 17:1–18. 10.1186/s12864-016-2612-7 27090510 PMC4835825

[74] McDougald L. Blackhead disease (histomoniasis) in poultry: a critical review. Avian Dis. (2005) 49:462–76. 10.1637/7420-081005R.1 16404985

[75] Adamu M Boonkaewwan C Gongruttananun N Vongpakorn M. Hematological, Biochemical and histopathological changes caused by coccidiosis in chickens. Agric Nat Resour. (2013) 47:238–46.

[76] Ruhnke I Andronicos NM Swick RA Hine B Sharma N Kheravii SK. Immune responses following experimental infection with *Ascaridia galli* and necrotic enteritis in broiler chickens. Avian Pathol. (2017) 46:602–9. 10.1080/03079457.2017.1330536 28503936

[77] Kim E-S Hong Y Lillehoj H. Genetic effects analysis of myeloid leukemia factor 2 and T cell receptor-B on resistance to coccidiosis in chickens. Poult Sci. (2010) 89:20–7. 10.3382/ps.2009-00351 20008798

[78] Lühken G Gauly M Kaufmann F Erhardt G. Association study in naturally infected helminth layers shows evidence for influence of interferon-gamma gene variants on *Ascaridia galli* worm burden. Vet Res. (2011) 42:1–7. 10.1186/1297-9716-42-84 21749701 PMC3150263

[79] Worley K Collet J Spurgin LG Cornwallis C Pizzari T Richardson DSJME. Heterozygosity and survival in red junglefowl. Mol Ecol. (2010) 19:3064–75. 10.1111/j.1365-294X.2010.04724.x 20618904

[80] Norup LR Dalgaard TS Pleidrup J Permin A Schou TW Jungersen G. Comparison of parasite-specific immunoglobulin levels in two chicken lines during sustained infection with *Ascaridia galli*. Vet Parasitol. (2013) 191:187–90. 10.1016/j.vetpar.2012.07.031 22981407

[81] Yang Q Whitmore MA Robinson K Lyu W Zhang G. Butyrate, forskolin, and lactose synergistically enhance disease resistance by inducing the expression of the genes involved in innate host defense and barrier function. Antibiotics. (2021) 10:1175. 10.3390/antibiotics10101175 34680756 PMC8532606

[82] Giansanti F Rossi P Massucci MT Botti D Antonini G Valenti P. Antiviral activity of ovotransferrin discloses an evolutionary strategy for the defensive activities of lactoferrin. Biochem Cell Biol. (2002) 80:125–30. 10.1139/o01-208 11908636

[83] Chang HM Ou-Yang RF Chen YT Chen CC. Productivity and some properties of immunoglobulin specific against streptococcus mutans serotype c in chicken egg yolk (IgY). J Agric Food Chem. (1999) 47:61–6. 10.1021/jf980153u 10563850

[84] Zhu W Zhang J He K Geng Z Chen XJPs. Proteomic analysis of fertilized egg yolk proteins during embryonic development. Poult Sci. (2020) 99:2775–84. 10.1016/j.psj.2019.12.056 32359615 PMC7597458

[85] Young D Fan MZ Mine Y. Egg yolk peptides up-regulate glutathione synthesis and antioxidant enzyme activities in a porcine model of intestinal oxidative stress. J Agric Food Chem. (2010) 58:7624–33. 10.1021/jf1011598 20540508

[86] Lim W Song G. Differential expression of vitelline membrane outer layer protein 1: hormonal regulation of expression in the oviduct and in ovarian carcinomas from laying hens. Mol Cell Endocrinol. (2015) 399:250–8. 10.1016/j.mce.2014.10.015 25458700

[87] Wicher KB Fries E. Haptoglobin, a hemoglobin-binding plasma protein, is present in bony fish and mammals but not in frog and chicken. Proc Nat Acad Sci. (2006) 103:4168–73. 10.1073/pnas.0508723103 16537503 PMC1449665

[88] Davalos A Miguel M Bartolome B Lopez-Fandino R. Antioxidant activity of peptides derived from egg white proteins by enzymatic hydrolysis. J Food Prot. (2004) 67:1939–44. 10.4315/0362-028X-67.9.1939 15453585

[89] Nakamura S Kato A. Kobayashi KJJoA, Chemistry F. Enhanced antioxidative effect of ovalbumin due to covalent binding of polysaccharides. J Agric Food Chem. (1992) 40:2033–7. 10.1021/jf00023a001

[90] Correnti C Clifton MC Abergel RJ Allred B Hoette TM Ruiz M. Galline Ex-FABP is an antibacterial siderocalin and a lysophosphatidic acid sensor functioning through dual ligand specificities. Structure. (2011) 19:1796–806. 10.1016/j.str.2011.09.019 22153502 PMC3240821

[91] Mahon MG Lindstedt KA Hermann M Nimpf J Schneider WJ. Multiple involvement of clusterin in chicken ovarian follicle development: binding to two oocyte-specific members of the low density lipoprotein receptor gene family. J Biol Chem. (1999) 274:4036–44. 10.1074/jbc.274.7.4036 9933595

[92] Liu Y Cheng Y Shan W Ma J Wang H Sun J. Chicken interferon regulatory factor 1 (IRF1) involved in antiviral innate immunity via regulating IFN-B production. Dev Comp Immunol. (2018) 88:77–82. 10.1016/j.dci.2018.07.003 29981306

[93] Kogut MH Chiang H-I Swaggerty CL Pevzner IY Zhou HJ. Gene expression analysis of toll-like receptor pathways in heterophils from genetic chicken lines that differ in their susceptibility to salmonella enteritidis. Front Genet. (2012) 3:121. 10.3389/fgene.2012.00121 22783275 PMC3389315

[94] Tsuchiya K Nakajima S Hosojima S Thi Nguyen D Hattori T Manh Le T. Caspase-1 initiates apoptosis in the absence of gasdermin D. Nat Commun. (2019) 10:1–19. 10.1038/s41467-019-09753-2 31064994 PMC6505044

[95] Yu J Nagasu H Murakami T Hoang H Broderick L Hoffman HM. Inflammasome activation leads to caspase-1–dependent mitochondrial damage and block of mitophagy. Proc Nat Acad Sci. (2014) 111:15514–9. 10.1073/pnas.1414859111 25313054 PMC4217429

[96] Suzuki T Franchi L Toma C Ashida H Ogawa M Yoshikawa Y. Differential regulation of caspase-1 activation, pyroptosis, and autophagy via Ipaf and ASC in shigella-infected macrophages. PLoS Pathog. (2007) 3:e111. 10.1371/journal.ppat.0030111 17696608 PMC1941748

[97] Hong Y Kim E-S Lillehoj H Lillehoj E Song K-DJ. Association of resistance to avian coccidiosis with single nucleotide polymorphisms in the zyxin gene. Poult Sci. (2009) 88:511–8. 10.3382/ps.2008-00344 19211519

[98] Krkavcová E Kreisinger J Hyánková L Hyršl P Javurková V. The hidden function of egg white antimicrobials: egg weight-dependent effects of avidin on avian embryo survival and hatchling phenotype. Biology Open. (2018) 7:bio031518. 10.1242/bio.031518 29540428 PMC5936061

[99] Wellman-Labadie O Picman J Hincke MJ. Comparative antibacterial activity of avian egg white protein extracts. Br Poult Sci. (2008) 49:125–32. 10.1080/00071660801938825 18409086

[100] Matulova M Rajova J Vlasatikova L Volf J Stepanova H Havlickova H. Characterization of chicken spleen transcriptome after infection with *Salmonella enterica*. serovar enteritidis. PLos ONE. (2012) 7:e48101. 10.1371/journal.pone.0048101 23094107 PMC3477135

[101] Sedger LM McDermott M. Tnf and Tnf-receptors: from mediators of cell death and inflammation to therapeutic giants–past, present and future. Cytokine Growth Factor Rev. (2014) 25:453–72. 10.1016/j.cytogfr.2014.07.016 25169849

[102] Falschlehner C Schaefer U Walczak HJI. Following trail's path in the immune system. Immunology. (2009) 127:145–54. 10.1111/j.1365-2567.2009.03058.x 19476510 PMC2691779

[103] Li M Lai P Chou Y Chi A Mi Y Khoo K. Protein tyrosine phosphatase PTPN3 inhibits lung cancer cell proliferation and migration by promoting EGFR endocytic degradation. Oncogene. (2015) 34:3791–803. 10.1038/onc.2014.312 25263444

[104] Liu H-C Kung H-J Fulton JE Morgan RW Cheng H. Growth hormone interacts with the Marek's disease virus SORF2 protein and is associated with disease resistance in chicken. Proc Natl Acad Sci U S A. (2001) 98:9203–8. 10.1073/pnas.161466898 11470922 PMC55398

[105] Awwad K Hu J Shi L Mangels N Abdel Malik R Zippel N. Role of secreted modular calcium-binding protein 1 (SMOC1) in transforming growth factor B signalling and angiogenesis. Cardiovasc Res. (2015) 106:284–94. 10.1093/cvr/cvv098 25750188

[106] Chen S Wang L Chen J Zhang L Wang S Goraya MU. Avian interferon-inducible transmembrane protein family effectively restricts avian tembusu virus infection. Front Microbiol. (2017) 8:672. 10.3389/fmicb.2017.00672 28473814 PMC5397487

[107] Asiamah CA Liu Y Ye R Pan Y Lu L-l Zou K. Polymorphism analysis and expression profile of the estrogen receptor 2 gene in leizhou black duck. Poult Sci. (2022) 101:101630. 10.1016/j.psj.2021.101630 35033905 PMC8762077

[108] Bello SF Adeola AC Nie QJ. The study of candidate genes in the improvement of egg production in ducks-a review. Poult Sci. (2022) 101:101850. 10.1016/j.psj.2022.101850 35544958 PMC9108513

[109] Kang L Zhang Y Zhang N Zang L Wang M Cui X. Identification of differentially expressed genes in ovaries of chicken attaining sexual maturity at different ages. Mol Biol Rep. (2012) 39:3037–45. 10.1007/s11033-011-1066-x 21691707

[110] Xu J Gao X Li X Ye Q Jebessa E Abdalla BA. Molecular characterization, expression profile of the FSHR gene and its association with egg production traits in muscovy duck. J Genet. (2017) 96:341–51. 10.1007/s12041-017-0783-x 28674235

[111] Ye Q Xu J Gao X Ouyang H Luo W Nie QJP. Associations of Igf2 and Drd2 polymorphisms with laying traits in muscovy duck. PeerJ. (2017) 5:e4083. 10.7717/peerj.4083 29181280 PMC5702507

[112] Lestari D Murtini S Ulupi N Sumantri C. Polymorphism and association of DMA gene with total IGY concentration and ND antibody titer in Ipb-D2 chicken line. Trop Anim Sci J. (2022) 45:1–8. 10.5398/tasj.2022.45.1.1

[113] Lee KP Anthony NB Orlowski SK Rhoads DD. SNP-based breeding for broiler resistance to ascites and evaluation of correlated production traits. Hereditas. (2022) 159:1–15. 10.1186/s41065-022-00228-x 35090566 PMC8796538

[114] Cubas-Gaona LL Diaz-Beneitez E Ciscar M Rodríguez JF Rodríguez DJ. Exacerbated apoptosis of cells infected with infectious bursal disease virus upon exposure to interferon alpha. J Virol. (2018) 92:e00364–18. 10.1128/JVI.00364-18 29540594 PMC5952143

[115] Xu S Xue C Li J Bi Y Cao YJ. Marek's disease virus type 1 microRNA miR-M3 suppresses cisplatin-induced apoptosis by targeting Smad2 of the transforming growth factor beta signal pathway. J Viol. (2011) 85:276–85. 10.1128/JVI.01392-10 20962090 PMC3014179

[116] Hu J Bumstead N Barrow P Sebastiani G Olien L Morgan K. Resistance to salmonellosis in the chicken is linked to NRAMP1 and TNC. Genome Res. (1997) 7:693–704. 10.1101/gr.7.7.693 9253598

[117] Liu W Kaiser M Lamont S. Natural resistance-associated macrophage protein 1 gene polymorphisms and response to vaccine against or challenge with *Salmonella enteritidis* in young chicks. Poult Sci. (2003) 82:259–66. 10.1093/ps/82.2.259 12619803

[118] Liu X-Q Wang F Jin J Zhou Y-G Ran J-S Feng Z-Q. Myd88 polymorphisms and association with susceptibility to *Salmonella pullorum*. BioMed Res Int. (2015) 2015:692973. 10.1155/2015/692973 26881204 PMC4735975

[119] Lee JT. Epigenetic regulation by long noncoding RNAs. Science. (2012) 338:1435–9. 10.1126/science.1231776 23239728

[120] Chen X Ali Abdalla B Li Z Nie QJL. Epigenetic regulation by non-coding RNAs in the avian immune system. Life. (2020) 10:148. 10.3390/life10080148 32806547 PMC7459779

[121] Tahira AC Kubrusly MS Faria MF Dazzani B Fonseca RS Maracaja-Coutinho V. Long noncoding intronic RNAs are differentially expressed in primary and metastatic pancreatic cancer. Mol Cancer. (2011) 10:1–19. 10.1186/1476-4598-10-141 22078386 PMC3225313

[122] Heward JA Lindsay MAJTii. Long non-coding RNAs in the regulation of the immune response. Trends Immunol. (2014) 35:408–19. 10.1016/j.it.2014.07.005 25113636 PMC7106471

[123] Riege K Hölzer M Klassert TE Barth E Bräuer J Collatz M. Massive effect on lncrnas in human monocytes during fungal and bacterial infections and in response to vitamins A and D. Sci Rep. (2017) 7:1–13. 10.1038/srep40598 28094339 PMC5240112

[124] Yu H Mi C Wang Q Dai G Zhang T Zhang G. Long noncoding RNA profiling reveals that LncRNA BTN3A2 inhibits the host inflammatory response to Eimeria tenella infection in chickens. Front Immunol. (2022) 13:891001. 10.3389/fimmu.2022.891001 36091044 PMC9452752

[125] Burnside J Bernberg E Anderson A Lu C Meyers BC Green PJ. Marek's disease virus encodes MicroRNAs that map to meq and the latency-associated transcript. J Virol. (2006) 80:8778–86. 10.1128/JVI.00831-06 16912324 PMC1563840

[126] Zhao P Li X-J Teng M Dang L Yu Z-H Chi J-Q. *In vivo* expression patterns of MicroRNAs of gallid herpesvirus 2 (Gahv-2) during the virus life cycle and development of marek's disease lymphomas. Virus Genes. (2015) 50:245–52. 10.1007/s11262-015-1167-z 25666057 PMC4381040

[127] Han B He Y Zhang L Ding Y Lian L Zhao C. Long intergenic non-coding RNA GALMD3 in chicken marek's disease. Sci Rep. (2017) 7:1–13. 10.1038/s41598-017-10900-2 28860661 PMC5579197

[128] Zhao C Li X Han B Qu L Liu C Song J. Gga-miR-130b-3p inhibits MSB1 cell proliferation, migration, invasion, and its downregulation in MD tumor is attributed to hypermethylation. Oncotarget. (2018) 9:24187. 10.18632/oncotarget.24679 29849932 PMC5966247

[129] Zhang X Yan Y Lei X Li A Zhang H Dai Z. Circular RNA alterations are involved in resistance to avian leukosis virus subgroup-j-induced tumor formation in chickens. Oncotarget. (2017) 8:34961. 10.18632/oncotarget.16442 28415618 PMC5471026

[130] Qiu L Chang G Bi Y Liu X. Chen G. Circular RNA and mRNA profiling reveal competing endogenous RNA networks during avian leukosis virus, subgroup J-induced tumorigenesis in chickens. PLoS ONE. (2018) 13:e0204931. 10.1371/journal.pone.0204931 30286182 PMC6171863

[131] Li H Ji J Xie Q Shang H Zhang H Xin X. Aberrant expression of liver microrna in chickens infected with subgroup J avian leukosis virus. Virus Res. (2012) 169:268–71. 10.1016/j.virusres.2012.07.003 22800510

[132] Wang Q Gao Y Ji X Qi X Qin L Gao H. Differential expression of micrornas in avian leukosis virus subgroup J-induced tumors. Vet Microbiol. (2013) 162:232–8. 10.1016/j.vetmic.2012.10.023 23157947

[133] Ji J Shang H Zhang H Li H Ma J Bi Y. Temporal changes of MicroRNA Gga-Let-7b and Gga-Let-7i expression in chickens challenged with subgroup J avian leukosis virus. Vet Res Commun. (2017) 41:219–26. 10.1007/s11259-017-9681-1 28190219

[134] Gordon JW Scangos GA Plotkin DJ Barbosa JA Ruddle FH. Genetic transformation of mouse embryos by microinjection of purified DNA. Proc Nat Acad Sci. (1980) 77:7380–4. 10.1073/pnas.77.12.7380 6261253 PMC350507

[135] Hammer RE Pursel VG Rexroad CE Wall RJ Bolt DJ Ebert KM. Production of transgenic rabbits, sheep and pigs by microinjection. Nature. (1985) 315:680–3. 10.1038/315680a0 3892305

[136] McGrew MJ Sherman A Ellard FM Lillico SG Gilhooley HJ Kingsman AJ. Efficient production of germline transgenic chickens using lentiviral vectors. EMBO Rep. (2004) 5:728–33. 10.1038/sj.embor.7400171 15192698 PMC1299092

[137] Zhu L Van de Lavoir M-C Albanese J Beenhouwer DO Cardarelli PM Cuison S. Production of human monoclonal antibody in eggs of chimeric chickens. Nat Biotechnol. (2005) 23:1159–69. 10.1038/nbt1132 16127450

[138] Schusser B Collarini EJ Yi H Izquierdo SM Fesler J Pedersen D. Immunoglobulin knockout chickens via efficient homologous recombination in primordial germ cells. Proc Nat Acad Sci. (2013) 110:20170–5. 10.1073/pnas.1317106110 24282302 PMC3864345

[139] Véron N Qu Z Kipen PA Hirst CE. Marcelle CJ. CRISPR mediated somatic cell genome engineering in the chicken. Dev Biol. (2015) 407:68–74. 10.1016/j.ydbio.2015.08.007 26277216

[140] Oishi I Yoshii K Miyahara D Kagami H Tagami TJ. Targeted mutagenesis in chicken using CRISPR/cas9 system. Sci Rep. (2016) 6:1–10. 10.1038/srep23980 27050479 PMC4822141

[141] Oishi I Yoshii K Miyahara D Tagami T. Efficient production of human interferon beta in the white of eggs from ovalbumin gene–targeted hens. Sci Rep. (2018) 8:1–12. 10.1038/s41598-018-28438-2 29976933 PMC6033876

[142] Kheimar A Klinger R Bertzbach LD Sid H Yu Y Conradie AM. A genetically engineered commercial chicken line is resistant to highly pathogenic Avian leukosis virus subgroup. J Microorganisms. (2021) 9:1066. 10.3390/microorganisms9051066 34069313 PMC8157034

[143] Koslová A Trefil P Mucksová J Reinišová M Plachý J Kalina J. Precise CRISPR/Cas9 editing of the nhe1 gene renders chickens resistant to the J subgroup of avian leukosis virus. Proc Nat Acad Sci. (2020) 117:2108–12. 10.1073/pnas.1913827117 31964810 PMC6995012

[144] Hellmich R Sid H Lengyel K Flisikowski K Schlickenrieder A Bartsch D. Acquiring resistance against a retroviral infection via CRISPR/Cas9 targeted genome editing in a commercial chicken line. Front Genome Ed. (2020) 2:3. 10.3389/fgeed.2020.00003 34713212 PMC8525359

[145] Challagulla A Jenkins KA O'Neil TE Shi S Morris KR Wise TG. *In Vivo* inhibition of Marek's disease virus in transgenic chickens expressing Cas9 and Grna against Icp4. Microorganisms. (2021) 9:164. 10.3390/microorganisms9010164 33450980 PMC7828426

[146] Koslová A Kučerová D Reinišová M Geryk J Trefil P Hejnar J. Genetic resistance to Avian leukosis viruses induced by CRISPR/Cas9 editing of specific receptor genes in chicken cells. Viruses. (2018) 10:605. 10.3390/v10110605 30400152 PMC6266994

[147] Rieblinger B Sid H Duda D Bozoglu T Klinger R Schlickenrieder A. Cas9-expressing chickens and pigs as resources for genome editing in livestock. Proc Nat Acad Sci. (2021) 118:e2022562118. 10.1073/pnas.2022562118 33658378 PMC7958376

[148] Tan FL Yin JQ. RNAi a new therapeutic strategy against viral infection. Cell Res. (2004) 14:460–6. 10.1038/sj.cr.7290248 15625012 PMC7092015

[149] Manjunath N Wu H Subramanya S Shankar P. Lentiviral delivery of short hairpin RNAs. Adv Drug Deliv Rev. (2009) 61:732–45. 10.1016/j.addr.2009.03.004 19341774 PMC2789654

[150] Zhang P Wang J Wan J Liu W. Screening efficient Sirnas *in vitro* as the candidate genes for chicken anti-avian influenza virus H5n1 breeding. Mol Biol. (2010) 44:37–44. 10.1134/S0026893310010061 32214469 PMC7089267

[151] Wang S Qu Z Huang Q Zhang J Lin S Yang Y. Application of gene editing technology in resistance breeding of livestock. Life. (2022) 12:1070. 10.3390/life12071070 35888158 PMC9325061

[152] Bello SF Xu H Guo L Li K Zheng M Xu Y. Hypothalamic and ovarian transcriptome profiling reveals potential candidate genes in low and high egg production of white muscovy ducks (*Cairina moschata*). Poult Sci. (2021) 100:101310. 10.1016/j.psj.2021.101310 34298381 PMC8322464

[153] Moon SH Lee JH Lee M Park E Ahn DU Paik HD. Cytotoxic and antigenotoxic activities of phosvitin from egg yolk. Poult Sci. (2014) 93:2103–7. 10.3382/ps.2013-03784 24902700

[154] Moon SH Lee JH Lee YJ Chang KH Paik JY Ahn DU. Screening for cytotoxic activity of ovotransferrin and its enzyme hydrolysates. Poult Sci. (2013) 92:424–34. 10.3382/ps.2012-02680 23300310

[155] Nassar FS. Poultry as an experimental animal model in medical research and pharmaceutical industry. Biomed J Sci Tech Res. (2018). 10.26717/BJSTR.2018.02.000751

[156] Boulland J-L Halasi G Kasumacic N Glover JCJJ. Xenotransplantation of human stem cells into the chicken embryo. J Vis Exp. (2010) 11:e2071. 10.3791/2071 20644515 PMC3144657

[157] Martowicz A Kern J Gunsilius E Untergasser GJJ. Establishment of a human multiple myeloma xenograft model in the chicken to study tumor growth, invasion and angiogenesis. J Vis Exp. (2015) 99:e52665. 10.3791/52665 25993267 PMC4542136

[158] Ching KH Collarini EJ Abdiche YN Bedinger D Pedersen D Izquierdo S. Chickens with humanized immunoglobulin genes generate antibodies with high affinity and broad epitope coverage to conserved targets. MAbs. (2018) 10:71–80. 10.1080/19420862.2017.1386825 29035625 PMC5800366

[159] Flemming Alexandra. Human Antibodies from Chicken Eggs. Nature Reviews Drug Discovery. (2005) 4:884-5. 10.1038/nrd1883

